# Structure, Dynamics, and Allosteric Potential of Ionotropic Glutamate Receptor N-Terminal Domains

**DOI:** 10.1016/j.bpj.2015.06.061

**Published:** 2015-08-06

**Authors:** James Krieger, Ivet Bahar, Ingo H. Greger

**Affiliations:** 1 Neurobiology Division, MRC Laboratory of Molecular Biology, Cambridge, UK; 2 Department of Computational and Systems Biology, School of Medicine, University of Pittsburgh, Pittsburgh, Pennsylvania

## Abstract

Ionotropic glutamate receptors (iGluRs) are tetrameric cation channels that mediate synaptic transmission and plasticity. They have a unique modular architecture with four domains: the intracellular C-terminal domain (CTD) that is involved in synaptic targeting, the transmembrane domain (TMD) that forms the ion channel, the membrane-proximal ligand-binding domain (LBD) that binds agonists such as L-glutamate, and the distal N-terminal domain (NTD), whose function is the least clear. The extracellular portion, comprised of the LBD and NTD, is loosely arranged, mediating complex allosteric regulation and providing a rich target for drug development. Here, we briefly review recent work on iGluR NTD structure and dynamics, and further explore the allosteric potential for the NTD in AMPA-type iGluRs using coarse-grained simulations. We also investigate mechanisms underlying the established NTD allostery in NMDA-type iGluRs, as well as the fold-related metabotropic glutamate and GABA_B_ receptors. We show that the clamshell motions intrinsically favored by the NTD bilobate fold are coupled to dimeric and higher-order rearrangements that impact the iGluR LBD and ultimately the TMD. Finally, we explore the dynamics of intact iGluRs and describe how it might affect receptor operation in a synaptic environment.

## Introduction

Ionotropic glutamate receptors (iGluRs) are glutamate-gated cation channels that mediate the majority of fast excitatory synaptic transmission in the central nervous system (1) and are key players in synaptic plasticity, a process that underlies learning and memory (2). iGluRs have also been implicated in various diseases and therefore are important drug targets (3, 4, 5, 6).

**Figure 1:**
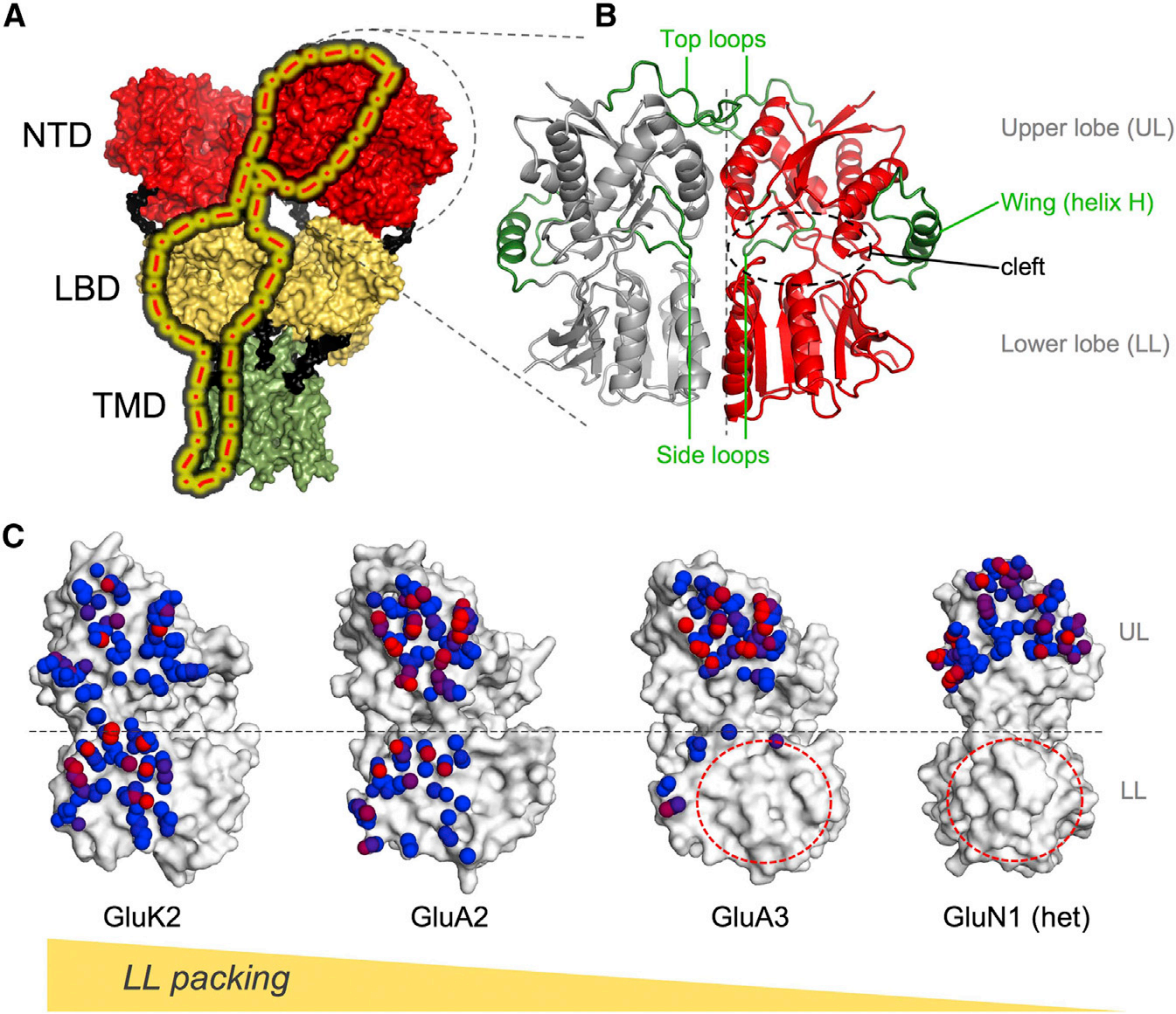
Structure of iGluR NTDs. (*A*) A structure of a homomeric GluA2 AMPAR (PDB ID: 3KG2) (18) is shown with the three main domain layers colored red (NTD), yellow (LBD), and green (TMD). The interdomain linkers are colored black. One subunit is highlighted. (*B*) A zoom-in of an NTD dimer illustrates the clamshell structure of each protomer (upper lobe (UL) and lower lobe (LL) separated by a cleft), as well as four features that vary between iGluRs: the overall dimeric packing, the top loop, the wing element, and the side loop. (*C*) The dimer interfaces of selected iGluR NTD dimers are shown as color-coded spheres for atoms forming contacts (interfacial atom-to-atom distance = <4.5 Å). Spheres are colored according to the number of contacts, from blue (*n* = 1) to red (*n* ≥ 7). A spectrum of LL packing is shown, comparing iGluR NTD dimers (*left to right*). The kainate receptors (exemplified by a GluK2 homodimer; PDB ID: 3H6H) (60) show the most LL packing similar to their UL packing (these interfaces show local contact densities (LDs) (100) of 27.0 and 35.1, respectively). Among the AMPAR paralogs, homodimeric LL packing correlates with affinity with GluA2 (PDB ID: 3H5V, dimer *AB*) (46) having the most tightly packed LLs (LD 23.4 vs. 43.5 for UL) and GluA3 (PDB ID: 3O21, dimer *CD*) (57) having minimal LL packing (LD 6.7 vs. 45.0 for UL). The NMDARs are at the far end of the spectrum, with no LL packing at all (shown for GluN1 of the heterodimer; PDB ID: 3QEL, dimer *AB*) (64). The dashed red circles in GluA3 and GluN1 highlight the lack of LL packing. A more detailed analysis of NTD dimer interfaces can be found in our recent studies (32, 56, 57). To see this figure in color, go online.

Four iGluR subtypes are distinguished based on pharmacology and sequence similarity (7): AMPA receptors (AMPARs; GluA1–4), kainate receptors (KARs; GluK1–5), NMDA receptors (NMDARs; GluN1, GluN2A–D, and GluN3A-B), and the orphan delta iGluRs (GluD1 and GluD2). iGluRs subunits have a modular structure with four domains (Fig. 1*A*) (1): the intracellular C-terminal domain (CTD), which is involved in synaptic targeting (8); the extracellular N-terminal domain (NTD); and the membrane-proximal ligand-binding domain (LBD) attached to the transmembrane domain (TMD; ion channel). The extracellular layers are connected to each other and to the TMD by peptide linkers, which are critical for interdomain communication and gating (9, 10, 11, 12, 13, 14, 15).

iGluRs assemble into homo- or heterotetramers, with a twofold symmetric extracellular region (ECR) and an ∼4-fold symmetric TMD. The ECR accounts for ∼80% of an iGluR and has a unique organization, with the distal NTDs forming two pairs of stable dimers that associate into tetramers via a small interface. Subunits cross over and swap dimeric partners at the level of the LBD with regard to the NTD (Fig. 1*A*) (16, 17, 18). This results in a loosely organized but at the same time interwoven ECR assembly, which will have key consequences on allosteric communication and ultimately on gating. Recent structural data show that both ECR layers can drastically reorganize in response to channel activation and desensitization (19, 20), confirming earlier findings (21). This notion is supported by a recent analysis of the collective motions that are accessible to intact AMPARs and NMDARs (22).

**Figure 2:**
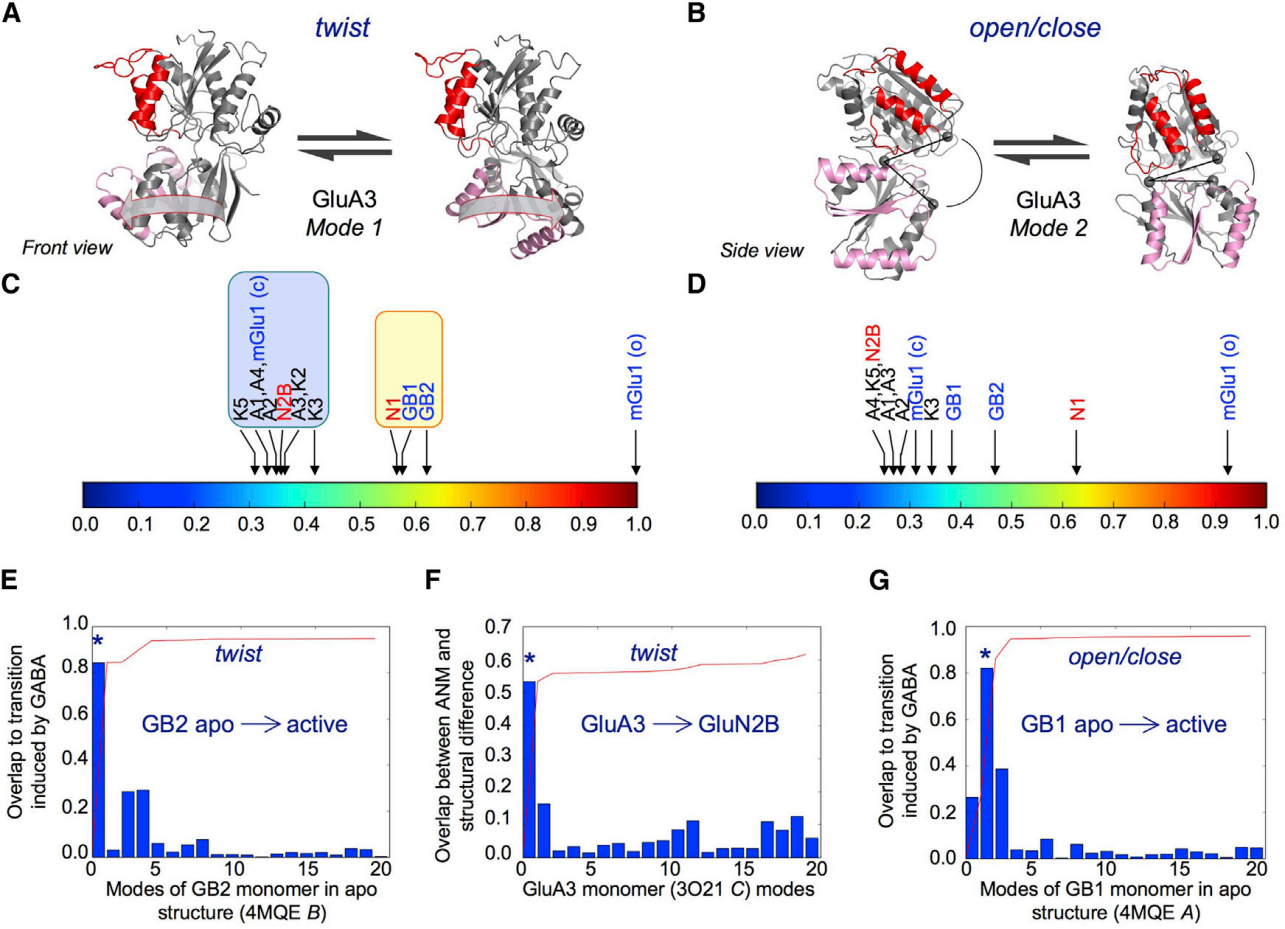
Motions accessible to iGluR NTD monomers. (*A*) Mode 1 is an interlobe twist. This is illustrated for AMPAR paralog GluA3 (PDB ID: 3O21, chain *C*) (57) using a front view based on the ULs (approximately constant position). The dimer interface is colored red (UL) and pink (LL). An arbitrary extent of motion is shown. (*B*) Mode 2 is a classical PBP cleft motion. A side view is shown with the cleft angle indicated by three marker residues (one in the UL, one in the hinge region, and one in the LL). Again the dimer interface is colored red and pink, and the extent of motion is arbitrary. (*C* and *D*) The extents of motion (square displacements) for the twisting motion (*C*) and cleft closure motion (*D*) are compared across iGluR NTDs (NMDARs in *red*) and the NTD-like allosteric modules from subunits of metabotropic glutamate (mGlu) and GABA_B_ (GB) receptors (both type C GPCRs; *blue*). The scale is relative to the twisting motion of an open mGlu1 clamshell (PDB ID: 1EWV) (30). The twisting motion (*C*) shows two clear clusters besides the open mGlu1, highlighted in blue and yellow. This segregation is less clear for the cleft motion (*D*). More details of the structures used for this analysis are listed in Table S1. (*E*) The ANM modes of the GB2 NTD-like module are compared against the conformational transition induced upon activation of the dimer by GABA binding to the GB1 subunit. Correlation cosines (or overlaps) are shown as blue bars. The red curve shows the cumulative overlap (root mean square of the correlation cosines). A twisting motion stands out (the asterisk marks mode 1). (*F*) The twisting motion (the asterisk marks mode 1) also stands out when we assess the overlaps between the ANM modes of the GluA3 NTD monomer and the difference between the structures of GluA3 and GluN2B NTD monomers. (*G*) A comparison of the ANM modes of the GB1 module against its conformational change upon GABA binding reveals a cleft closure motion (the asterisk marks mode 2).

The iGluR NTD and LBD, both of which belong to the periplasmic binding protein (PBP) superfamily, have bilobate structures that evolved to bind ligands in the interlobe cleft (see Fig. 2*B*) (23). The NMDAR NTD is believed to function like a classic PBP allosteric module that is stabilized in a closed-cleft conformation upon binding of the cleft ligand zinc, which triggers a reduced channel open probability (10, 24, 25). Related dimeric, allosterically active type I PBPs are found in natriuretic peptide receptors (NPRs) (26, 27, 28) and type C G-protein-coupled receptors (GPCRs), including metabotropic glutamate receptors (mGluRs) and the GABA_B_ receptor (29, 30).

To date, a signaling role has not yet been described for the NTD in non-NMDARs (AMPARs and KARs), where this domain forms tight dimers and is mostly involved in subtype-selective subunit assembly (31, 32). In addition to tighter dimeric packing, reduced allosteric activity in non-NMDARs is also suggested by the overall ECR architecture, which is loosely organized. In the more tightly packed NMDAR (16, 17), extensive interactions between NTD and LBD permit allosteric coupling and hence changes in the channel open probability in response to NTD ligands (6). Nevertheless, the flexible ECR assembly in non-NMDARs permits dynamic rearrangements, which appears to facilitate interactions with other synaptic components, including auxiliary subunits in *cis* (9) and perhaps other proteins in *trans*, i.e., via presynaptic interactions (33, 34, 35), as has been described for GluDs (36, 37). These reconfigurations could drastically alter the allosteric landscape of non-NMDARs and their anchorage/diffusion at synapses.

To provide a better understanding of the iGluR ECR, a strategic drug target, we revisit NTD dynamics at the level of the monomer, dimer, and tetramer, as well as whole-receptor dynamics. We review recent data on NTD structure/dynamics and further elaborate on NTD dynamics at the level of the monomer, the dimer, and the intact receptor tetramer using coarse-grained and all-atom molecular-dynamics (MD) simulations. Motions are benchmarked against the fold-related mGluR and GABA_B_ ligand-binding cores (LBCs), which we also investigate in this study.

## Materials and Methods

**Figure 3:**
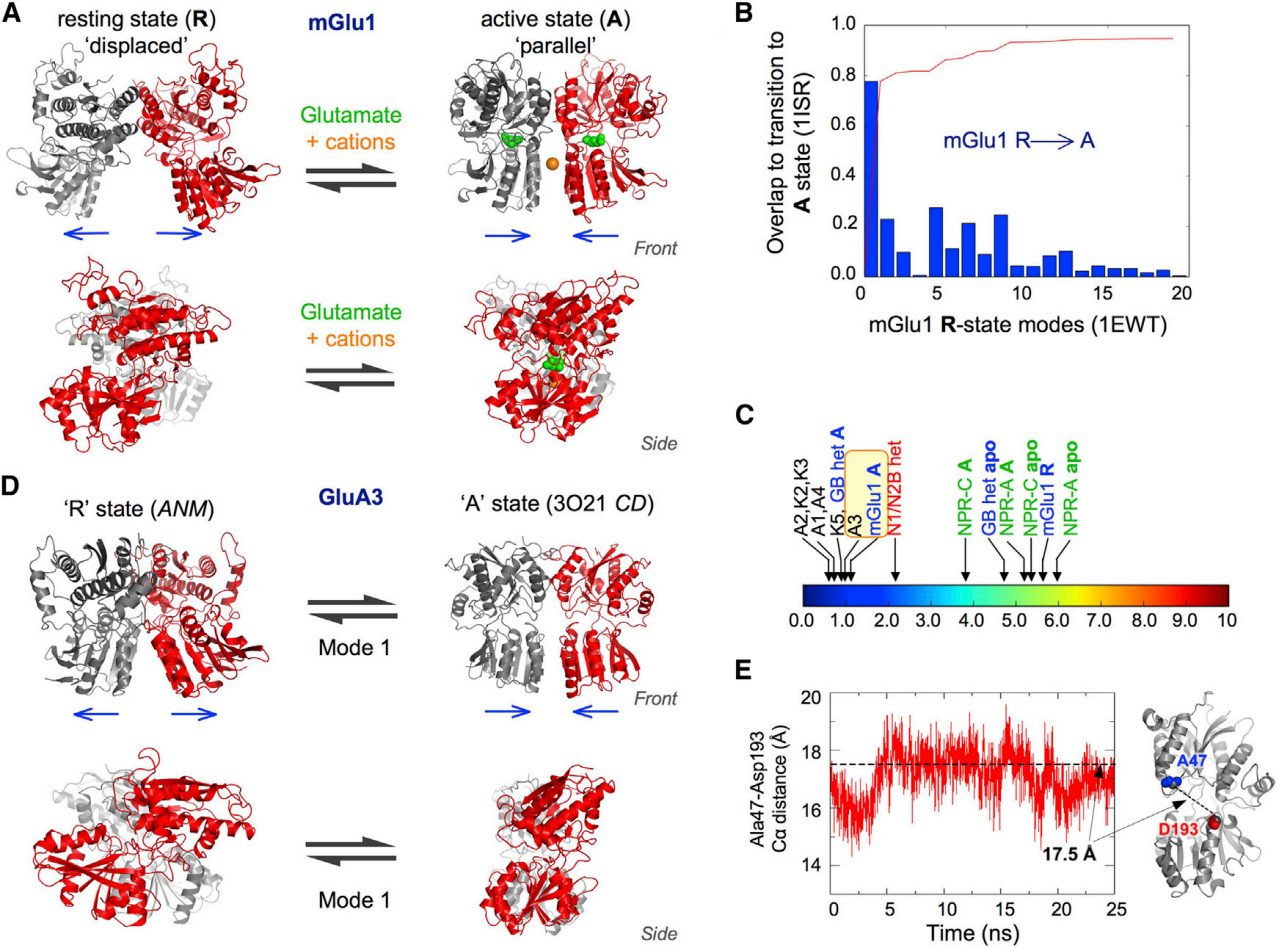
Motions that are accessible to dimers. (*A*) mGluRs have been captured in a displaced resting (R) state (exemplified by PDB ID: 1EWT) (30) and a parallel active (A) state (stabilized by glutamate and cations, *green and orange spheres*; PDB ID: 1ISR) (82). Front and side views are shown. (*B*) These structures can interconvert with mode 1 (an interprotomer counterrotation), accounting for most of the transition. The blue bars and red curve are overlaps and cumulative overlaps, respectively, as in Fig. 2. (*C*) The extent of this motion is compared across NTDs relative to the monomer motions (mGlu1 twisting = 1). The GluA3 AMPAR NTD homodimer stands out as a result of its decreased LL packing (similar to state A mGlu1, *yellow*). More details of the structures used for this analysis are listed in Table S1. (*D*) Right: GluA3 was captured in a conformation resembling the mGluR A state. Left: ANM mode 1 enables the GluA3 NTD dimer to reach a conformation resembling the R state. (*E*) NTD dimers also exhibit cleft motions in the ANM (56) and in all-atom MD simulations. Variations in the C*α* distance between A47 and D193 in simulations of a GluA2 NTD dimer (PDB ID: 3H5V) (46) reveal an interlobe twist similar to that of monomer ANM Mode 1. The first 25 ns portion of one simulation is shown for this distance in one subunit. The horizontal stippled line denotes the 17.5 Å C*α* distance seen in the crystal structure. The GluA2 NTD structure on the right shows the position of the marker residues (A47, *blue*; D193, *red*) and the distance between their C*α* residues (*stippled line between dark gray spheres*).

The anisotropic network model (ANM) treats each residue as a node at the C*α* position, with interactions within a cutoff distance of 15 Å modeled as springs using harmonic potentials with uniform spring constants for all interacting residues (38). The ANM yields a unique spectrum of modes for each examined structure, ranging from low-frequency (or soft) modes to high-frequency (or stiff) modes. Each mode *k* (3*N*-6 of them for a structure of *N* residues) is characterized by a shape (3*N*-dimensional eigenvector *u*_*k*_) and frequency (eigenvalue *λ*_*k*_) determined by eigenvalue decomposition of the 3*N* × 3*N* Hessian matrix H. Mode 1 is the softest mode (with the smallest eigenvalue, or effective force constant), mode 2 is the next softest, and so on. Soft modes are usually highly distributive, as illustrated in Movies S3, S4, and S5. The modes at the other end of the spectrum are highly localized. To generate a conformation along a given mode *k*, we add the quantity [± *aλ*_*k*_^−1/2^*u*_*k*_] to the 3*N*-dimensional position vector *R* composed of the original C*α* coordinates, where the arbitrary coefficient *a* is varied to generate the movies as described earlier (39). We chose these multiplication factors so that we could clearly illustrate each motion. We used the ANM web server (40) and PyMOL (41) when creating the movies and the panels containing structures shown in Figs. 2, 3, and 5 and Fig. S2 in the Supporting Material.

The amplitude of motion for a given mode is taken as the square displacements along that mode that scale with *λ*_*k*_^−1^. As such, we were able to compare the relative mode amplitudes by comparing the eigenvalues. All comparisons in this work (Figs. 2, *C* and *D*, and 3*C*) were made relative to the interlobe twisting motions of an open mGlu1 clamshell (PDB ID: 1EWV) (30). The normalized amplitude of each ANM mode (in arbitrary units) was taken as a fraction of the normalized amplitude of mGlu1 mode 1 by dividing mGlu1 eigenvector 1 by the eigenvector of the mode with which it was compared. Long N- and C-terminal extensions were cleaned when necessary. The structures used are listed in Table S1 along with their eigenvalues and relative amplitudes.

**Figure 4:**
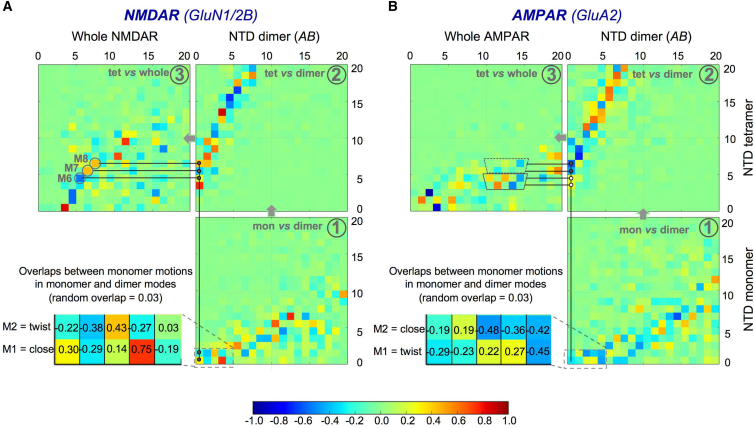
(*A* and *B*) Monomer and dimer conformational changes are coupled to motions of NTD tetramers and whole receptors in both NMDARs (*A*) and AMPARs (*B*). Matrices comparing the modes of motion of these smaller systems with progressively larger systems are represented as colored squares. Darker colors indicate higher correlations. Red (positive correlations) and blue (negative correlations) are equivalent, as ANM modes are harmonic fluctuations with arbitrary starting directions. The bottom matrix (*panel 1*) shows the overlap between modes of monomer motions (ordinate, label on *far right* and shared by AMPARs and NMDARs) and dimer motions (abscissa). A zoom into the first few elements illustrates that many dimer modes show a significant overlap with both prominent modes of monomer motion. The matrix above (*panel 2*) compares the same dimer modes of motion (shared abscissa) with the modes of motion of the NTD tetramer (ordinate, label on *far right*). In this case, there are many darker blocks, indicating a higher correlation. The top-left matrix shows the overlap between the motions of the NTD tetramer and those of the whole receptor. The starting structure for the AMPAR is a GluA2 homotetramer (18); the NMDAR is a GluN1/2B heteromer (PDB ID: 4PE5) (16). Lines connect some modes of motion that show good correlations all the way from monomers to whole receptors. Dimeric motions are more conserved through the levels in the NMDAR, as illustrated for dimer mode 1, where dark dots illustrate good correlations along the way and the endpoints are the circled modes 6–8 of the whole NMDAR. The monomer shown is GluN1, where cleft motions are also well retained. In the AMPAR monomer, cleft motions are again retained but dimer rearrangements are dampened in the whole receptor. This results in poor correlations transitioning from the NTD tetramer to the whole receptor (*white dots* and *stippled trapezium*). Also evident is a higher dominance of tetramer motions in lower-frequency modes of the NMDAR, where the tighter ECR packing restricts rearrangements in which the NTD moves as a rigid body.

To compare the ANM modes with experimentally observed structural differences (Figs. 2, *E–G*, 3*B*, and S2 B), we first calculated the vector corresponding to the structural differences (displacements of corresponding C*α* atoms) and then calculated the correlation cosine (or overlap) between this vector and each ANM mode vector. Cumulative overlaps are defined as the square root of the sum of correlation cosines squared. In a similar way, we compared ANM modes between smaller and larger systems (Fig. 4). We used subsets of the mode vectors for the large systems that corresponded to only those residues found in the relevant small systems. We ran all calculations in Python using the ProDy package (42).

We carried out all-atom MD simulations using GROMACS 4.6 (43) and the CHARMM27 force field (44). Bonds that contained hydrogen atoms were constrained with LINCS (45), allowing 2 fs time steps. The starting structure was an isolated NTD dimer (PDB ID: 3H5V) (46), which was imbedded in a rectangular box extending at least 9 Å away from the protein in any direction. This was filled with TIP3P water molecules (47), which were then randomly replaced with sodium and chloride ions to neutralize the system and reach a concentration of 70 mM (a total of 53 sodium and 49 chloride ions). Two rounds of steepest-descent energy minimization (5000 steps), NVT equilibration (1 ns), and NPT equilibration (1 ns) were performed before production MD. The first round included restraints on the protein heavy atoms to equilibrate the solvent around the protein. The temperature was set at 300 K by means of a stochastic velocity rescaling thermostat (48) throughout. The pressure was set at 1 bar using the Berendsen barostat (49) during equilibration and the Parrinello-Rahman barostat (50) during production MD. Short-range noncovalent interactions were cut off at 12 Å using the particle-mesh Ewald method for long-range electrostatics (51), and van der Waals interactions were smoothly switched off between 10 Å and 12 Å. This protocol was carried out for two independent replicas, each lasting 100 ns.

**Figure 5:**
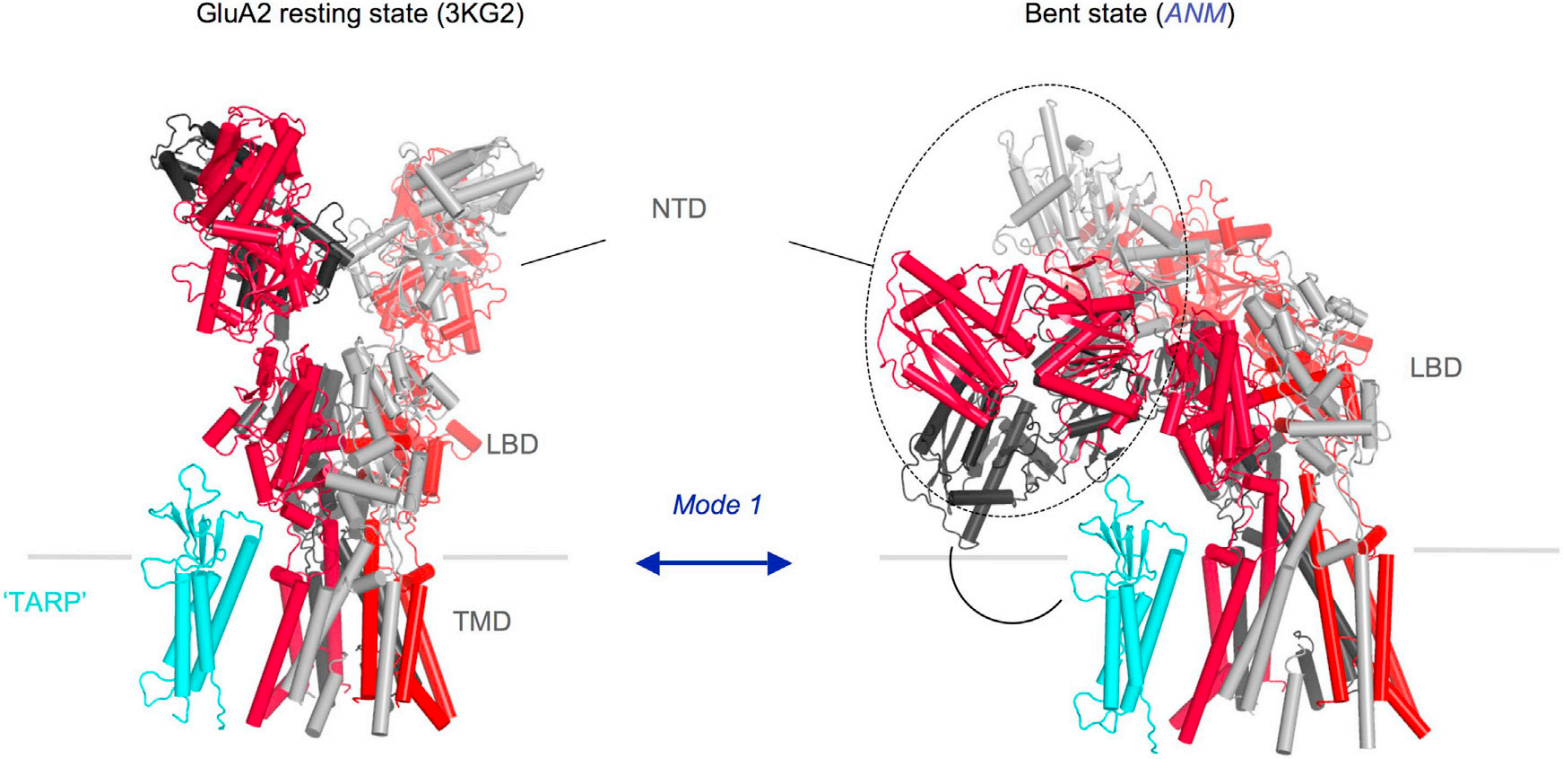
ANM of the whole GluA2 AMPAR shows global bending motions that could bring the NTD into proximity with auxiliary subunits such as TARPs. An AMPAR crystal structure (PDB ID: 3KG2) (18) is shown on the left, with the two nonequivalent chain pairs colored in red (distal from the NTD tetramer interface) and gray (proximal and interface forming). A homology model of TARP *γ*-2 based on the related claudins is shown in cyan. The structure on the right shows a conformation along ANM mode 1 in which the NTD bends down and can contact the TARP. To see this figure in color, go online.

The homology model of TARP *γ*-2 shown in Fig. 5 was generated using MODELLER (52). Template selection and alignment were carried out using the HHPred server (53, 54).

## Results

### iGluR NTD structures: differences and similarities

NTD crystal structures are available for all three main iGluR subtypes. These include homodimers of all four AMPAR paralogs (46, 55, 56, 57, 58), KAR homodimers (GluK2, GluK3, and GluK5), and a heterodimer (GluK2/GluK5) (59, 60, 61), as well as NMDAR GluN1 and GluN2B as homo- and heterodimers (62, 63, 64). NMDAR NTD homodimers do not form in solution (64) and NMDARs function exclusively as heteromers (65). These high-resolution structures can also be interpreted in the context of intact AMPARs, NMDARs, and KARs at lower resolution (16, 17, 18, 19, 20, 66, 67, 68).

iGluR NTDs have a PBP-like clamshell structure with a cleft separating the upper lobe (UL) and lower lobe (LL) (Figs. 1*B* and 2*B*), which are connected via three peptide hinges (a characteristic of type I PBPs) (69). NTDs show an intermediate cleft angle compared with other, allosterically active PBPs (38), which is similar across all AMPARs (56) and KARs (59, 60). Current structures of NMDAR NTDs appear to be in a closed-clamshell conformation, in both the absence and presence of the GluN2 cleft ligand Zn^2+^ (62, 63); hence, cleft motions have only been inferred from simulations (56, 70) and have not been observed crystallographically.

A number of differences among the AMPAR, KAR, and allosterically active NMDAR NTDs are evident. At the level of the monomers, the following features stand out (Fig. 1*B*). 1) The NMDAR NTD lobes are uniquely twisted (62, 63, 71), creating a coordination site for zinc. The GluK5 NTD also exhibits an interlobe twist relative to the other AMPARs and KARs (59), although to a much lesser extent. 2) A wing-like helical extension of hinge 2 is seen in AMPAR and KAR NTDs. The AMPAR NTD wing is highly dynamic in all-atom MD simulations (56) and thus may be of functional relevance. This segment is absent in NMDARs, which likely permits increased intraprotomer dynamics. 3) A loop facing the dimer interface varies in length, and this side loop is shortest in the AMPARs. The side loop projects into the dimer interface in KARs and has been suggested to affect receptor assembly (60). 4) Loop variation is also apparent at the top of the protomer, with this top loop, or flap, being longest in AMPARs, also suggested to play a role in subfamily-selective assembly (46, 55, 60, 72). Collectively, these features likely impact intra- and interprotomer dynamics, which are described below.

Similarly to other receptor families that harbor NTD-like modules (26, 27, 29, 30), iGluR NTD dimers may be the functional unit, i.e., ligands binding to monomers will result in dimer rearrangements that in turn trigger allosteric signal transmission. NTDs assemble into dimers with their clefts facing in opposite directions, and, as noted above, dimeric packing differs between iGluR subfamilies. AMPAR and KAR NTDs exhibit more extensive associations involving both the UL and LL, whereas NMDAR dimers are mostly held together via their ULs, which will facilitate intradimer motions (Fig. 1*C*). AMPAR NTD dimers generally show tighter packing between the ULs, as evidenced by their higher local atomic contact density (LD) (24) (quantified in Fig. 1*C* and previous studies (32, 56, 57)). This feature contrasts with KAR NTD dimers, which exhibit more uniform LDs between the ULs and LLs (32). Within the AMPAR subfamily, differences are mostly seen across the variable LL dimer interface, with GluA2 and GluA3 at the extremes showing extensive and minimal LL packing, respectively (Fig. 1*C*) (32, 56), in line with dimer affinities measured by analytical ultracentrifugation (61, 64, 72, 73).

The looser organization of NMDAR NTDs with unconstrained, mGluR-like LLs renders the dimer more dynamic, likely underlying their role in allosteric gating regulation (6, 64). The constraint of LL mobility observed in early AMPAR and KAR NTD structures ruled out NMDAR-like allostery (46, 59, 60, 61, 63, 64). This picture changed with the GluA3 NTD structure (PDB ID: 3O21) (57), which revealed relatively unconstrained LL contacts resulting from like-charge repulsion between arginines. Moreover, simulations using both coarse-grained and all-atom models revealed unexpected flexibility within NTD dimers (56, 57), as we documented with additional simulations described below.

### iGluR NTD monomers can undergo classical PBP cleft motions

To elucidate the dynamic spectrum of iGluR NTDs, we utilized all-atom MD simulations and elastic network model calculations. In particular, we used the ANM, an elastic network model in which each residue is represented as a node centered at its C*α* position with springs connecting interacting residues (74, 75). The resulting harmonic potential can be solved analytically to determine all possible motions, which are decomposed into a series of normal modes. The first, energetically favorable (low-frequency) modes describe correlated, global motions, whereas high-frequency modes (denoted by higher mode numbers) represent localized motions. Previous ANM analyses provided first insights into the dynamics of the AMPAR and NMDAR NTDs (56, 57, 70). Here, we extend those analyses by comparing these NTDs with the other non-NMDAR subfamily, KAR, as well as the related LBCs of mGluRs and GABA_B_ receptors.

The two most accessible global modes at the level of NTD monomers exhibit interlobe twisting (Fig. 2, *A* and *C*) and opening/closing motions of the clamshell cleft (Fig. 2, *B* and *D*) (56, 57). These NTD cleft motions are observed to various degrees, presumably depending on the specific interactions that constrain the hinges connecting the two lobes. They are also apparent in the related mGluRs (30) and the iGluR LBDs (type II PBPs) (76). Although no cleft ligand has been identified yet for non-NMDAR NTD clefts, electron density has been observed in GluA2 (55, 57) and GluK2 (60). Interlobe twisting has been described for NMDAR NTDs (70, 71) and also in the GluA2 LBD, where it has been linked to partial agonist binding (77).

In line with our previous ANM analysis of AMPAR and NMDAR NTDs (56), we find that both modes of motion are conserved across the iGluRs, but the extent varies (Fig. 2, *C* and *D*). For example, both the low-affinity KARs (GluK2 and K3) and the high-affinity KAR (GluK5) are very similar to AMPARs (*red* and *blue blocks* in Fig. S1*, A* and *B*). There are, however, subtle differences due to an extension of the wing toward the LL, especially in the low-affinity KARs, causing the twisting mode to exhibit a cleft-closure component. To benchmark motions in iGluR NTDs and to aid comparison, we analyzed the dynamics of the related modules from mGluRs (mGlu1 subunit in open and closed conformations) and GABA_B_ receptors (GB1 and GB2 subunits), which are allosterically active receptor PBPs that have been crystallized in various ligand-bound states (29, 30, 78, 79).

Despite its large wing element, the twisting motion is largest for the open mGlu1 monomer, and the remaining structures are shown relative to this value (Fig. 2*C*; Table S1). Most iGluR NTDs (including GluN2B) cluster together on the extent scale, with the exception of GluN1, which aligns more closely with the GABA_B_ modules (*yellow cluster*). GABA_B_ receptors may indeed use a twist motion for activation. We find that a rotation of the GB2 LL toward the dimer interface is apparent when the apo and GABA-bound structures are superposed (PDB ID: 4MQE and 4MS3) (29). The same two conformations are also observed in crystal structures of GB2 extracellular domain alone (78). We next assessed how well this twisting of GB2 corresponds to its ANM modes. For this purpose, we first calculated the vector of differences for the C*α* position of each corresponding GB2 residue between the two heterodimeric structures. Next, we calculated the overlap between this vector and each ANM mode vector as a correlation cosine (*blue bars* in Fig. 2*E*). This analysis indeed showed that ANM mode 1 of GB2 from the apo structure (*asterisk* in Fig. 2*E*) could account for the difference between these two crystallographically observed GB2 conformations. Interestingly, GluN2B, where twisting has been observed structurally (63), and which has been linked to function (71), clusters with the AMPAR and KAR NTDs (Fig. 2*C*, *blue cluster*). Hence, AMPAR and KAR NTD protomers also have capacity to twist and untwist to an extent similar to that observed for GluN2B. Accordingly, we find that AMPAR and KAR NTDs have the intrinsic ability to twist toward the conformation seen in GluN2B via ANM mode 1 (shown for GluA3 in Fig. 2*F*).

Classic PBP cleft motions (30) are also observed for all NTD-like protomers analyzed, with the degree of motion again illustrated relative to mGlu1 twisting (Fig. 2*D*; Table S1). We can see that the extent and overall order of cleft motions are generally comparable to the twisting. However, we observe no clear segregation between the known allosteric modules (i.e., NMDA and GABA_B_) and non-NMDA iGluRs at this level. Moreover, GB2 (which is not believed to bind ligands) has the ability to undergo cleft motions similar to those observed for the ligand-coordinating GB1 subunit, which closes upon GABA binding (*asterisks* in Fig. 2*G*) (29). This may be linked to transmission of the signal of agonist binding from the GB1 cleft to the G-protein-binding GB2 subunit, which is required for receptor activation (80, 81).

In summary, our analysis reveals that NTD (and NTD-like) monomers of these receptors share two major types of intraprotomer motions, twisting and cleft opening/closure, consistent with the intrinsic flexibility of the type I PBP fold that they share. The analysis further shows that these modular structures do not exhibit discrete states (or switches between these states) but rather a continuum of conformations along the mode coordinates. The extent of motion is defined by the specific sequence (or side-chain interactions) of the particular receptor or by the ligands (e.g., Zn^2+^) that stabilize particular conformers, whereas the global hinge mechanism is robustly retained. These intraprotomer motions are coupled to dimeric rearrangements as described in the next section.

### iGluR NTD dimers undergo rearrangements similar to those observed in mGluRs

Dimeric rearrangements of NTD-like modules in response to ligand are believed to trigger allosteric signal transduction in type C GPCRs. This has been documented in mGluRs, where ligand-free NTD dimers appear to exist in equilibrium between displaced and parallel dimer conformations, both of which were captured by crystallization (Fig. 3*A*) (30) and were apparent in previous ANM simulations (57). The more parallel conformation is favored in the agonist-bound, closed-cleft state (the active (A) state) and the displaced conformation (the resting (R) state) (Fig. 3*A*) (30). These states are characterized by an intradimer rotation mediated by the UL dimer interface. Negatively charged residues at the LL dimer interface couple the cleft conformation to the dimeric state: the A state is unstable with both clefts open and can only be obtained with at least one cleft closed (30). Cations can bind and stabilize this LL dimer interface, enabling the closure of both clefts (82) and maximal receptor activity (83). This cation modulation has been observed in both heterologously expressed mGluRs and neurons (84). As noted previously (57), a single ANM mode (the first and thus lowest-energy mode, M1), which features an interprotomer counterrotation, accounts for most of the transition between the R and A states (Fig. 3*B*). These findings highlight an interplay between cleft motions and the dimer configuration.

Analogous dimer rotations are evident in ANM simulations of GABA_B_ extracellular domain dimers (mode 1; Fig. S2 A). However, the crystal structures of this domain (both apo and GABA bound) contain parallel dimers (29); the major difference between the two states appears to be a closure of the LL dimer interface. A comparison of the GABA-induced conformation change with ANM modes accessible to the apo form (Fig. S2 B) shows that activation involves both dimer rotation (albeit small) and LL closure (mode 2), as well as intraprotomer conformational changes (twisting and cleft closure), which are seen to various extents in higher-frequency modes 3–8. This analysis highlights parallels and differences in the dynamic spectra of these closely related structures.

Dynamics related to mGluR1 can be inferred from GluN1/2B NTD heterodimeric structures (64). The NMDAR NTD dimer can access a similar mode of interprotomer counterrotation to reach a parallel, A-like state (Fig. S2*C*) (56). In this case, the A state could be stabilized by polyamines (e.g., spermine), positive allosteric modulators that bind to the LL dimer interface to alleviate like-charge repulsion (24). Contrary to the case in mGluRs, it has been suggested that in NMDARs the A state is coupled to an open cleft and that cleft closure drives it to the R state (resulting in negative modulation). This prediction is confirmed by our ANM analysis (Fig. S2*C*; Movie S1), which shows that cleft motions are apparent in GluN1 but reduced in GluN2B. This is readily explained by the formation of an interface between the GluN2B LL and the GluN1 UL (restricting GluN2B cleft motions). The heterodimer is trapped in an R-like, displaced state by the NMDAR-negative allosteric modulator ifenprodil, which binds to the UL dimer interface (64). Ifenprodil derivatives hold promise for the development of subtype-selective NMDAR-negative modulators.

Related dimer rotations are also accessible to AMPAR (57) and KAR NTDs, despite their more extensive dimer associations. This is most evident for GluA3, which stands out from other AMPARs and KARs in a map of relative mobilities (Fig. 3, *C* and *D*; see also Table S1) due to its looser LL packing (Fig. 1*D*) (56, 57). The dynamics of GluA3 NTDs can be explained by their substantially weaker dimer associations (72) that uniquely give rise to a variety of dimeric arrangements in GluA3 NTD crystal structures (57) (I.H.G., J. Garcia, and B. Herguedas, unpublished data). The loosely packed GluA3 conformation resembles the mGluR A state (57), with a root mean-squared deviation in the positions of the corresponding C*α* atoms of ∼5 Å (when comparing the 3O21 dimer *CD* with 1ISR and 1EWK). This arrangement is likely to be unstable due to like-charge repulsion of the LLs in the absence of a stabilizing anion, analogous to the situation for mGluRs and cations (57). The functional consequence of this unique behavior in GluA3, relative to the other AMPAR NTD paralogs, remains to be determined.

We observe that dimer rotations are coupled to concerted intraprotomer twisting and cleft motions in mode 1 of all AMPARs and KARs, as well as the agonist-bound GABA_B_ module, which features tighter LL associations (compared with the apo form). In particular, the LLs twist upward (closing the cleft) and toward the dimer interface as the protomers become displaced (R-like) (shown for GluA2 in Movie S2). Thus, the AMPAR and KAR NTD dimers approach an NMDAR-like structure in which the LL of one subunit contacts the UL of the other. In addition to showing the same motion as the AMPAR NTD dimers in this mode, the dimeric KAR NTD behavior is very similar to that of the AMPARs up to ∼15 ANM modes (Fig. S1 C). The GluK5 KAR NTD homodimer has an intermediate structure along this mode, showing greater intraprotomer twisting and interprotomer displacement relative to the other AMPARs and KARs. The retention of intraprotomer modes of motion after dimerization was also apparent in our previous analysis of AMPAR NTD dynamics (56). This coupling likely results from the packing between LLs, and cleft motions are reduced as LL associations loosen up (for a given extent of rotation). As in previous all-atom MD simulations of AMPAR NTDs (56), we observed cleft twisting in our all-atom MD simulations of a GluA2 dimer (PDB ID: 3H5V), especially during the first 10 ns, during which time the LL of one chain twisted toward the dimer interface, thereby decreasing the distance between D193 and A47 to ∼14 Å (compared with 17.5 Å in the crystal structure; Fig. 3*E*).

In addition to enabling dimer rotations and clamshell motions (56, 57), the looser packing in the GluA3 NTD homodimer also permits a splaying apart of the LLs, which has been observed in all-atom MD simulations (56). This motion resembles that observed in NPRs, where NTD-like dimers also exhibit an opening between LLs, and closure is triggered via binding of a cyclic peptide (natriuretic peptide) to the LL dimer interface (26). Protomer counterrotations have also been observed in NPR crystal structures (27, 28), and our ANM analysis shows that both LL interface closure and protomer counterrotations are important for peptide-induced conformational changes in both NPR-A and NPR-C (data not shown). LL dimer interface opening and closure are also apparent in an ANM analysis of GluA3 (mode 4), the A state mGluR1 (mode 3), and the GABA_B_ apo form (mode 2 discussed above).

Overall, a common theme for receptors harboring type I PBP dimeric modules emerges in which intraprotomer cleft motions are coupled to displacement and LL closure of NTD-like dimers. This set of motions appears to form a principal pathway of signal propagation for these diverse receptor families. Next, we examine how dimer dynamics are transmitted within the tetrameric iGluR ECR.

### Monomer and dimer motions are coupled to larger rearrangements in NTD tetramers and intact iGluRs

NTD dimers associate via a small interface (∼400 Å) in AMPARs and KARs. These tetramer contacts are mediated by the LLs and are apparent in some isolated NTD structures (46, 55, 59, 60, 61) as well as in intact receptors (18, 19, 20, 66, 67, 68). Helices engaged in this interface (*α*F and *α*G in GluA2) show structural heterogeneity (57) and are highly dynamic in solution when analyzed at the single-molecule level (85) as well as in all-atom MD simulations (56). Together with the global motions described above, these local fluctuations could impact the formation, stability, and dynamics of the NTD layer. NMDAR NTD dimers appear to come together via an interface involving related segments in the GluN2B LL, although the overall arrangement of the NTD layer diverges (16, 17).

We recently studied the global dynamics of whole AMPARs and NMDARs, and found unexpected similarities between the two iGluRs (22). We observed some conservation of NTD tetramer and dimer dynamics in intact iGluRs (22), which, together with the finding that NTD monomer dynamics are conserved in the dimer (28), suggests that these motions persist in whole receptors. Here, to unravel allosteric communication in iGluR ECRs, we focused on this hierarchy of motions, i.e., how monomer and dimer dynamics relate to motions in NTD tetramers and whole receptors. For this purpose, we performed a stepwise comparison of smaller and larger systems, i.e., we compared NTD monomers with dimers, NTD dimers with tetramers, and the isolated NTD tetramer layer with whole receptors. These comparisons are depicted in correlation matrices arranged end to end for the level that comprises a large system in one and a small system in the next (*gray arrows*, Fig. 4). Each matrix element represents the overlap between the directions of motion of the selected small system in isolation and in the context of the larger system that contains it, for a given ANM mode of each system. We did not perform this analysis on KARs because no sufficiently complete whole-receptor structure is available and KAR NTD tetramers behave the same as AMPAR NTD tetramers (Fig. S1*D*).

First, we investigated the interplay of ECR dynamics in NMDAR, which is of functional consequence (6). In this receptor, the two ECR layers interdigitate, which presumably restricts their dynamics (22) but at the same time forms an allosteric unit (16, 17). Localized motions in the NTD layer, such as cleft opening/twisting in the protomers and dimeric rotations, are indeed still apparent and are affected by the packing within and between ECR layers. As outlined in Fig. 4*A* (and detailed in the legend), we observe significant conservation of the two dominant monomer motions (Fig. 2, *A* and *B*) in the first global modes of the dimers (Fig. 4*A*, *panel 1* and *zoom*) as indicated by red and blue elements, which represent high correlations (positive and negative overlaps, respectively; this sign is not relevant, as ANM modes are harmonic fluctuations with arbitrary starting directions). At the next level, dimer motions are retained in isolated tetramers (Fig. 4*A*, *panel 2*), and the tetramer modes of motion that contain these also show good correlations in the whole receptor (Fig. 4*A*, *panel 3*).

Whole-receptor modes 6–8 (Fig. 4*A*, *panel 3*, M6–M8) are of particular interest because they feature NMDAR clamshell dynamics. As shown above, these are more pronounced in GluN1 and less so in GluN2B. GluN2B is known to bind Zn^2+^ ions, which trigger an allosteric cascade, ultimately resulting in a decreased channel open probability (15, 86). Zn^2+^ has been suggested to stabilize the closed-cleft conformation to initiate this negative modulation (6, 10, 24, 63, 86). Focusing on mode 6, we observe that GluN1 cleft closure triggers a displaced R-like state, whereas GluN1 cleft opening is coupled to the dimer adopting a more parallel, A-like conformation (Movie S3; Fig. S2 C). This state could be stabilized by polyamine binding between the LLs, which would potentiate the channel (24). Dimer rotations are associated with substantial reorientations of the NTD layer and the entire ECR. Approximation of the dimer A state is accompanied by a hinging at the tetramer interface that results in a more upright NTD layer that makes less contact with LBD, as well as a stretching of the LBD-TMD linkers. Related to this, our recent study (22) showed that modes related to mode 6 bring the NMDAR toward an AMPAR-like structure, highlighting the close similarities in the dynamic spectra of the two subfamilies. Conversely, the transition toward the R state results in a flipping toward a horizontal NTD layer, with the GluN2B subunits pushing down on the LBD (Movie S3), which could explain the finding that zinc binding to the NTD destabilizes the LBD dimers, resulting in a desensitized-like state (86).

For the GluA2 AMPAR, NTD clamshell motions are also evident in isolated NTD tetramers and the whole receptor (Fig. 4*B*). Like NMDAR, AMPAR shows a clear conservation of monomer dynamics in the dimers and dimer motions in the NTD tetramer (compare correlations in Fig. 4, *A* and *B*, *panels 1* and *2*). However, in the whole AMPAR (*panel 3*), these are found in higher ANM modes, which are characterized by more localized (high-frequency) motions. This may result from the looser packing of the AMPAR ECR, which enables a greater variety of global rearrangements (see below) (22). Another difference is that in AMPAR, NTD dimer rotations can be discerned in the isolated NTD tetramer but are reduced in the intact receptor. This likely results from the tighter LL packing of AMPAR (GluA2) NTD dimers. This difference is illustrated in Fig. 4*B* (*panels 2* and *3*), where higher tetramer modes that correlate well with the dimer (*dark dots* in *panel 2*) exhibit lower overlaps in the whole receptor (*top trapezium* in *panel 3*), whereas tetramer modes that correlate poorly with those of the dimer (*white dots* in *panel 2*) show higher overlaps in the whole receptor (*bottom trapezium* in *panel 3*).

Surprisingly, two modes of NTD tetrameric motion dominate the dynamics of the intact AMPAR (each correlating well with multiple whole AMPAR modes): 1) NTD dimer sliding (tread-climb mode, e.g., mode 11; Movie S4) and 2) rotation of the NTD dimers toward each other (e.g., mode 12; Movie S5). These motions resemble those seen in NMDAR modes 6–8 and are likewise accompanied by an approximation of the NTD and LBD at one extreme of the motion. Unlike the case with NMDAR, in AMPAR the NTD dimers are approximately symmetric, with both clefts equally able to undergo clamshell motions. The NTDs that form the tetramer interface (i.e., the inner or proximal chains, equivalent to GluN2) are coupled to hinging of the NTD tetramer interface, resulting in increased cleft motions in these chains. A clear cleft opening/closure component is seen in mode 11, whereas a twisting motion dominates in mode 12. Whether this capacity for intrinsic motions in the AMPAR ECR is related to allosteric communication remains to be elucidated.

### Large-scale AMPAR NTD rearrangements, global bending motions, and interactions with synaptic components

In addition to internal rearrangements, including those described above, the AMPAR tetramer interface can also rupture to enable rearrangements associated with receptor desensitization (19, 20, 21). In KARs, which have an ECR organization closely resembling that of AMPAR, the NTD tetramers do not seem to dissociate (20, 67), which may be due to the greater stability of the KAR tetrameric interface (K_d_ = ∼5 *μ*M) (61) compared with AMPAR NTD tetramers (at least 10-fold weaker) (46, 55, 72, 73). The relative mobilities in the first few ANM modes of GluK2 and GluA2 NTD tetramers are consistent with the relative stability of the GluK2 versus GluA2 ECRs (data not shown).

At excitatory synapses, iGluRs are in close proximity to a multitude of pre- and postsynaptic components, which may vary in a synapse-specific fashion. Large-scale receptor motions could impact interactions with synaptic proteins. Non-NMDARs are also associated with auxiliary subunits, both secreted and membrane bound, many of which have been identified for AMPARs (87, 88, 89, 90) and modulate their trafficking and gating (91). We recently showed that transmembrane AMPAR regulatory proteins (TARPs) can interact with the NTD in vitro (9). A cross talk between the membrane-distal NTD with the membrane-proximal TARP would require substantial movements of the ECR, resulting in receptor reconfiguration, which could impact receptor function, diffusion, and ultimately synapse organization. Bending motions, which would facilitate such interactions, are indeed apparent in AMPAR and NMDAR ANM. In fact, the first and hence highest-amplitude mode of the intact AMPAR features this motion (Fig. 5). We note that the energy/frequency of such large bending events cannot be accurately predicted from the ANM as a result of the use of uniform spring constants. Conformational changes within the NTD layer and global rearrangements of the ECR may also have an impact on interactions with N-cadherin (34), secreted pentraxins (33, 35), and other, yet to be identified components. This cross talk may even be related to the reported synaptogenic role of the GluA2 NTD (92, 93, 94), a function that is well established for the related GluD1 and GluD2 NTDs (36, 37). Allosteric regulation of synaptic iGluRs is a complex, currently ill-explored topic.

## Conclusion

iGluRs have a unique architecture among other ligand-gated channels, which is characterized by a large, modular ECR (accounting for 80% of the receptor mass). The ECR is made up of eight PBP-like clamshell domains that are connected via flexible linkers between the ECR layers and are associated via interfaces, featuring a range of affinities, within the layers (72, 73, 95). This architecture offers a multitude of motions and sites for drug action, such as the clamshell cleft, dimer and tetramer interfaces, and interdomain regions connected by linkers. Here, we analyzed the NTD modules in a hierarchical manner by examining their monomeric, dimeric, tetrameric, and whole-receptor dynamics using existing crystal structures in combination with the ANM (75) and all-atom MD simulations. In line with earlier studies (22), we found differences and similarities between the NMDAR and AMPAR ECR dynamic spectra at all hierarchical levels.

At the monomer level, interlobe twisting and opening/closure motions are seen across the spectrum of NTD-like modules (Fig. 2). Surprisingly, twisting, which was first observed in GluN2B, is widely seen and therefore may not be unique to NMDAR NTDs. In fact, the ability to undergo this motion is equally accessible to AMPAR and KAR NTDs, and is prominent in mGluRs despite their large wing element. The wing is mostly packed against the UL but exhibits contacts (predominantly water mediated) with the LL in AMPARs, a structural element that is missing in NMDAR NTDs. No clashes are apparent upon visual examination of these AMPAR ANM modes, suggesting that the wing does not restrict these motions. At the level of the dimer, dominant motions include interprotomer rotations and opening of the LL dimer interface. These two themes are seen to various extents and levels across the examined structures, and are likely a core mechanism of signal transmission in receptors containing NTD-like signaling modules. For example, in the GABA_B_ receptor, the LL dimer opening/closing motion dominates with a smaller rotational component. Not surprisingly, we generally observe that large-scale motions in isolated systems (i.e., a PBP-like monomer) are restricted in larger systems to various extents. Nevertheless, motions of individual clamshells, including opening/closing and lobe twisting, are still apparent in intact receptors, where they are expected to trigger complex allosteric pathways that ultimately control channel gating.

Several key questions remain to be answered. Are the signs of non-NMDAR NTD dynamics functionally relevant or is the NTD in these iGluR subfamilies an orphan PBP that lost its signaling function (a scenario that is somewhat related to inactive enzymes (96, 97))? In this case, the sole role of the NTD would be restricted to subunit assembly (72, 98) and synapse organization/synaptogenesis (33, 92). How is allosteric communication via the NMDAR NTDs transmitted? Specifically, does Zn^2+^ binding trigger GluN2B NTD cleft closure followed by (large-scale) dimeric rearrangements (as shown in Figs. S2 C and Fig. 3) or are localized changes surrounding the Zn^2+^ coordination site (His-127 and Glu-284 in GluN2B) propagated to the dimer interface and the GluN1 NTD? We find that the GluN1 NTD exhibits large cleft motions, consistent with GluN1 allosteric activity (71). Moreover, the GluN2 NTD-LBD linkers, which have been shown to mediate NTD signaling, are packed between the NTD and LBD layers, forming an interface as revealed in current crystal structures (16, 17). How this interface and the NTD-LBD linkers, particularly of GluN2B (10, 15), mediate NTD signaling remains to be elucidated. The rapid progress that is being made in electron cryo-microscopy (20, 99) is expected to increase the suite of intact iGluR structures and reveal conformational intermediates. These will serve as substrates for further simulations to ultimately reveal the complex allosteric routes through these large signaling machines.

## Author Contributions

I.H.G. and J.K. designed the research. J.K. performed research and analyzed data. I.B. contributed analytical tools. J.K., I.B., and I.H.G. wrote the manuscript.

## Supporting Material

Document S1. Two figures and one table

Movie S1. GluN1/2B dimer Mode 1 showing a GluN1 cleft opening and closure coupled to dimer rearrangements GluN1 is red and GluN2B is gray. The extent of motion was chosen to give a parallel, closed A-like state that could bind polyamines. It should be noted that GluN2B cleft motions are limited by the interaction formed between its LL and the GluN1 UL in the ifenprodil-bound starting structure. Based on functional data (31,71), we expect that it would in fact open and close in concert with GluN1.

Movie S2. GluA2 dimer Mode 1 showing a cleft opening/closure and twisting coupled to dimer rearrangements A front view is shown, giving a view into the cleft of the gray subunit. The ANM provides information on the directionality of motions, but not on their absolute size; therefore, the extent of motion is arbitrary.

Movie S3. Whole NMDAR Mode 6 showing GluN1 NTD cleft motions coupled to larger rearrangements GluN1 subunits are in two reds and GluN2B subunits are in two grays. As noted in the legend for Movie S1, GluN2B cleft motions are limited in the ANM but are still expected to occur. This mode was found to be similar to AMPAR mode 7 in our most recent study (22) (see http://www.ccbb.pitt.edu/bahar/mw1.html). The ANM provides information on the directionality of motions, but not on their absolute size; therefore, the extent of motion in the movie is arbitrary. Here, the fluctuation amplitudes were selected to approximate the extent of conformational variations observed between the AMPAR and NMDAR, and to allow a clear visualization of the NTD conformational changes.

Movie S4. Whole AMPAR Mode 11 showing NTD cleft motions coupled to larger rearrangements Distal subunits (A/C) are in two reds and proximal subunits (B/D) are in two grays. As noted in the legend to Movie S3, the extent of motion shown is somewhat arbitrary.

Movie S5. Whole AMPAR Mode 12 showing NTD cleft motions coupled to larger rearrangements Subunits are colored as in Movie S4. As noted in the legend to Movie S3, the extent of motion is somewhat arbitrary.

Document S2. Article plus Supporting Material
