# Supplementary material for: Structure, Dynamics, and Allosteric Potential of Ionotropic Glutamate Receptor N-Terminal Domains
Source: Biophys J. 2015 Aug 6;109(6):1136–48. doi: 10.1016/j.bpj.2015.06.061 (PMC4576161; doi:10.1016/j.bpj.2015.06.061)
Supplement: Document S1. Two figures and one table [file mmc1.pdf]

Biophysical Journal

Supporting Material

Structure, Dynamics, and Allosteric Potential of Ionotropic Glutamate Receptor N-Terminal Domains

James Krieger,[1] Ivet Bahar,[2] and Ingo H. Greger[1,*]

[1]Department of Neurobiology, MRC Laboratory of Molecular Biology, Cambridge, UK; and [2]Department of Computational and Systems Biology, School of Medicine, University of Pittsburgh, Pittsburgh, Pennsylvania

Module	State [a]	PDB id	ref	Monomer [b, d]	Dimer [b, c]	Relative amplitude (eigenvalue) [c, d]		
						**Inter-lobe twist**	**Cleft opening / closure**	**Inter-protomer counter-rotation**
GluA1		3SAJ	(45)	B	BD	0.33 (0.36)	0.27 (0.43)	0.71 (0.164)
GluA2		3H5V	(25)	B	AB	0.35 (0.33)	0.28 (0.41)	0.60 (0.194)
GluA3		3O21	(26)	C	CD	0.37 (0.32)	0.27 (0.43)	0.98 (0.120)
GluA4		4GPA	(28)	-	sym	0.33 (0.36)	0.25 (0.47)	0.68 (0.171)
GluK2		3H6H	(23)	A	AB	0.36 (0.33)	0.30 (0.39)	0.61 (0.193)
GluK3		3OLZ	(46)	A	AB	0.41 (0.28)	0.34 (0.35)	0.52 (0.226)
GluK5		3OM0		-	sym	0.31 (0.38)	0.25 (0.45)	0.84 (0.139)
GluN1	**R-like** (ifenprodil inhibited)	3QEL	(27)	A	AB	0.56 (0.21)	0.63 (0.18)	2.16 (0.054)
GluN2B				B	AB	0.36 (0.33)	0.25 (0.46)	
mGlu1	**R**oo (apo I)	1EWT	(37)	B	AB	1.00 (0.12)	0.93 (0.13)	5.68 (0.021)
	**A**co (apo II)	1EWV		A	AB	0.33 (0.35)	0.32 (0.37)	2.28 (0.051)
	**A**cc (L-glu + gadolinium)	1ISR	(68)	-	sym	0.31 (0.37)	0.31 (0.38)	1.11 (0.106)
GB1	(apo)	4MQE	(36)	A	AB	0.57 (0.20)	0.38 (0.31)	4.77 (0.025)
GB2	(apo)			B		0.62 (0.19)	0.47 (0.25)	
GB	(GABA)	4MS3		A	AB	0.28 (0.42)	0.21 (0.56)	0.85 (0.138)
				B		0.51 (0.23)	0.45 (0.26)	
NPR-A	(apo)	1DP4	(101)		Aa			5.99 (0.020)
	**A-like** (ANP)	1T34	(35)		AB			5.22 (0.022)
NPR-C	(apo)	1JDN	(33)		sym			5.39 (0.022)
	**A-like** (CNP)	1JDP			AB			3.84 (0.031)

Table S1: Structures used for ANM calculations, relative square displacements and eigenvectors. Relative amplitudes and eigenvalues have arbitrary units.

a. Bold ‘R’ and ‘A’ refer to presumed resting and active dimeric conformations of mGluRs. Lower case letters indicate whether the mGluR clefts are open or closed. Closure of both clefts yields conformation **A**cc for full activation – this requires stabilisation by cations.

b. A hyphen indicates that there is only one protomer in the asymmetric unit. The dimer is generated from crystal symmetry (‘sym’). In the case where multiple subunits are found in the asymmetric unit but do not make up the dimer, symmetry-related subunits are represented by lower case letters.

c. NMDARs and GBRs are obligate heteromers. Values for heterodimers are given in merged cells.

d. Natriuretic peptide receptor protomers do not appear to predominantly use either of the intra-protomer motions described here and hence their amplitudes for these motions are not presented.

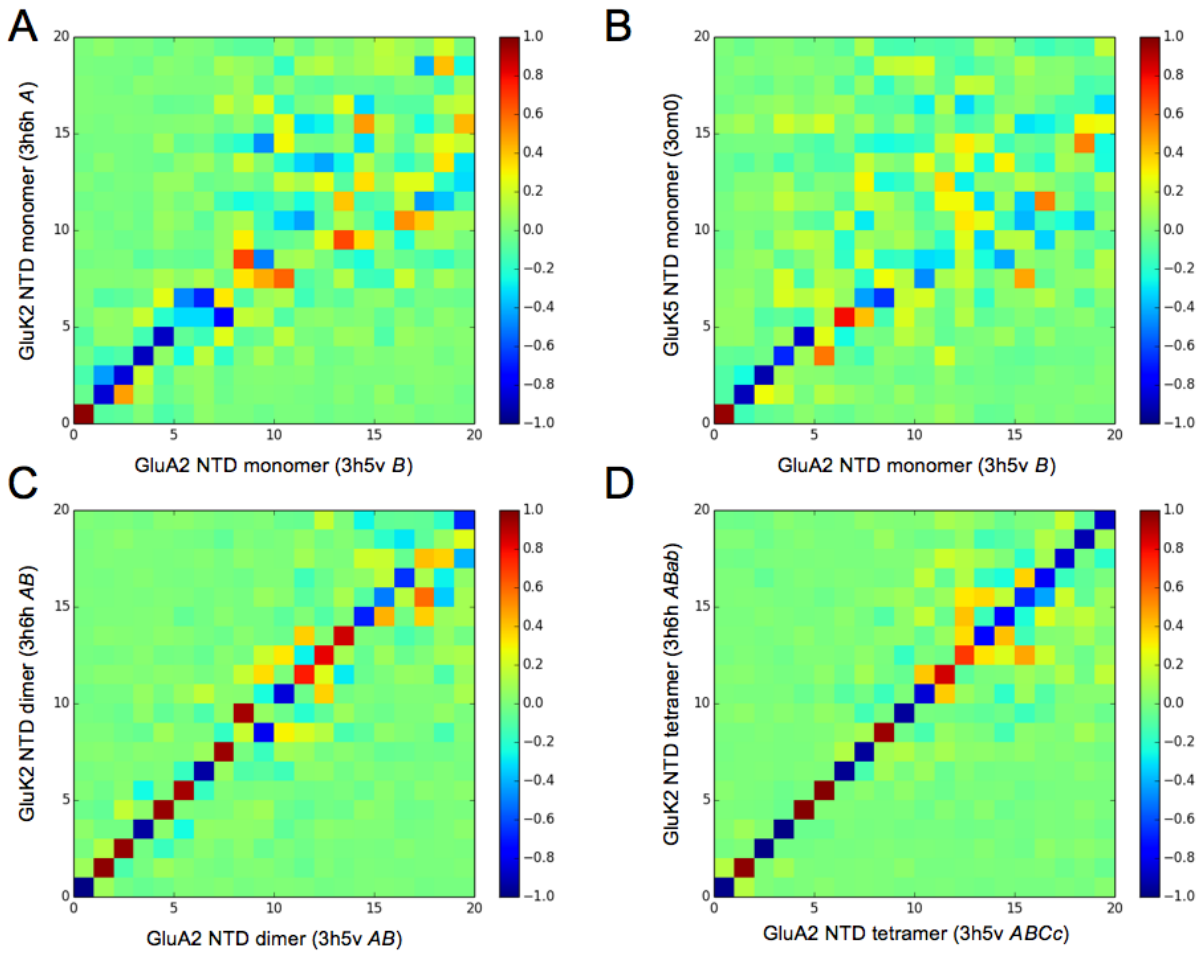

Supplementary Figure 1: Comparison of AMPAR and KAR NTD dynamics. **(A)** The first 20 global modes of a representative AMPAR NTD monomer (chain *B* from GluA2 structure 3H5V) are compared to those of a representative low-affinity KAR (chain *A* from GluK2 structure 3H6H). Red and blue blocks represent high positive and negative overlaps (equivalent as ANM modes have arbitrary starting direction). **(B)** The same AMPAR modes are compared against those of a representative high-affinity KAR (GluK5 structure 3OM0). **(C)** The first 20 global modes of an AMPAR NTD dimer (*AB* from GluA2 structure 3H5V) are compared to those of a representative KAR (GluK2 structure 3H6H). **(D)** The equivalent comparison is given for GluA2 and GluK2 NTD tetramers. Chains extracted from crystallographic symmetry are written in lower case.

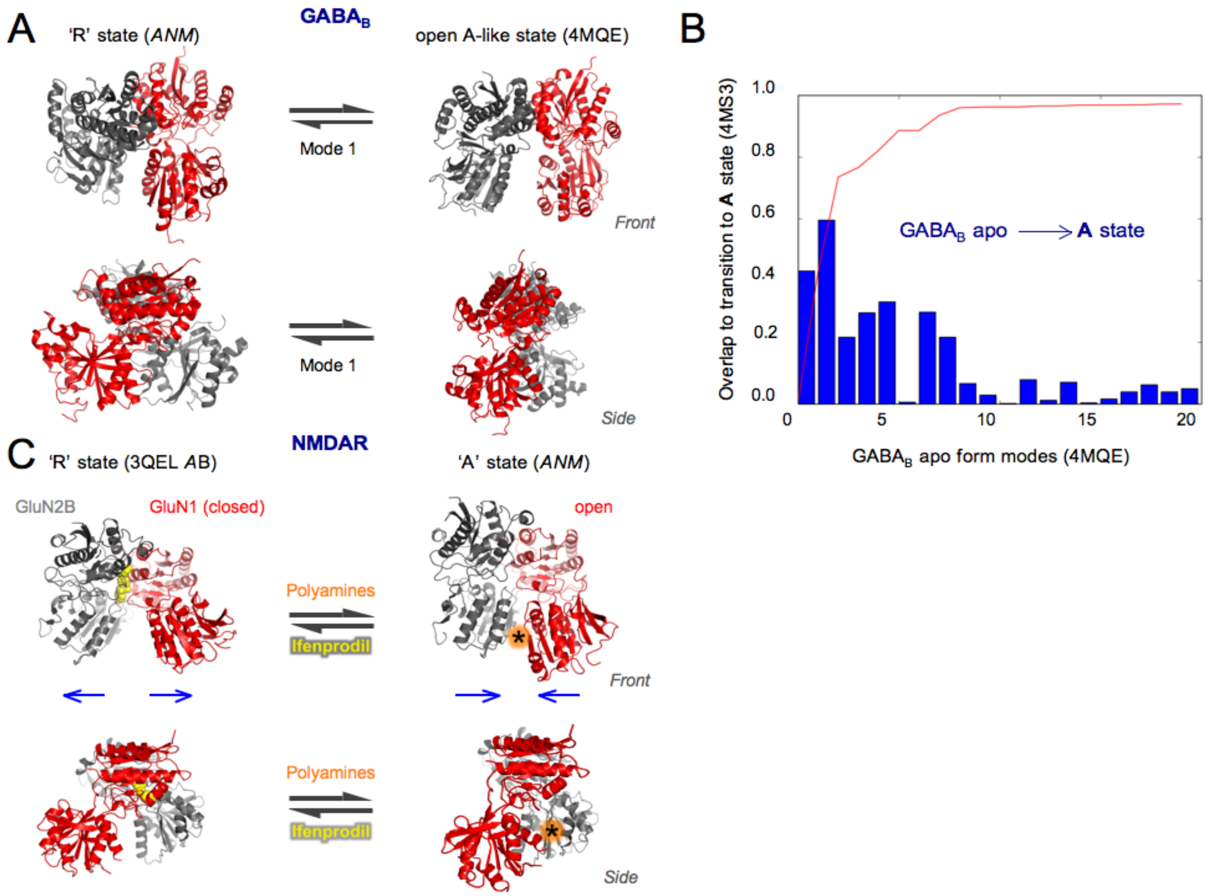

Supplementary Figure 2: Structure and dynamics of the GABA$_B$ receptor NTD-like module and the NMDA receptor NTD. **(A)** The GABA$_B$ receptor NTD-like module has been trapped in parallel conformations resembling the mGluR active (A) state even in the apo form (*right*). The major difference between them is that the apo (and antagonist-bound) structures exhibit more opening of the lower lobe dimer interface. ANM mode 1 of these structures still allows transitions to more displaced structures resembling the mGluR resting (R) state (*left*). **(B)** Comparison of the ANM mode vectors to the difference between the apo structure and the agonist-bound structure (A state) reveals that both mode 1 (intra-dimer rotation) and mode 2 (lower lobe closure) are important for GABA$_B$ receptor activation. **(C)** ANM mode 1 of the GluN1/2B heterodimer is also an interprotomer counter-rotation (see Fig. 3) (26, 28). The left panels show the crystal structure with the allosteric inhibitor ifenprodil bound (yellow), which traps the dimer in a state similar to the mGluR resting (R) state. The panel on the right shows a structure resembling the mGluR active (A) state, which is accessible *via* ANM mode 1.

Movie 1: GluN1/2B dimer mode 1 showing GluN1 cleft opening and closure coupled to the dimer rearrangements. GluN1 is red and GluN2B is grey. The extent of motion was chosen to give a parallel, closed A-like state that could bind polyamines. It should be noted that GluN2B cleft motions are limited by the interaction formed between its LL and the GluN1 UL in the ifenprodil-bound starting structure. We expect from functional data (31, 56) that it would in fact open and close in concert with GluN1.

Movie 2: GluA2 dimer mode 1 showing cleft opening/closure and twisting coupled to the dimer rearrangements. A front view is shown giving a view into the cleft of the grey subunit. The ANM provides information on the directionality of motions, but not on their absolute size. Therefore the extent of motion is arbitrary.

Movie 3: whole NMDAR mode 6 showing GluN1 NTD cleft motions coupled to larger rearrangements. GluN1 subunits are in two reds and GluN2B subunits two greys. As noted in the legend for Movie 1, GluN2B cleft motions are limited in the ANM but are expected to occur too. This mode was found to be similar to AMPAR mode 7 in our most recent study (22) (see http://www.ccbb.pitt.edu/bahar/mw1.html). The ANM provides information on the directionality of motions, but not on their absolute size. Therefore the extent of motions in the movies is arbitrary. The fluctuation amplitudes have been selected here to approximate the extent of conformational variations observed between the AMPAR and NMDAR, and to allow a clear visualization of the NTD conformational changes.

Movie 4: whole AMPAR mode 11 showing NTD cleft motions coupled to larger rearrangements. Distal subunits (A/C) are in two reds and proximal subunits (B/D) in two greys. As noted in the legend to Movie 3, the extent of motion shown is somewhat arbitrary.

Movie 5: whole AMPAR mode 12 showing NTD cleft motions coupled to larger rearrangements. Subunits are coloured as in Movie 4. As noted in the legend to Movie 3, the extent of motion is somewhat arbitrary.
