# Supplementary figures and images for: Structure, Dynamics, and Allosteric Potential of Ionotropic Glutamate Receptor N-Terminal Domains
Source: Biophys J. 2015 Aug 6;109(6):1136–48. doi: 10.1016/j.bpj.2015.06.061 (PMC4576161; doi:10.1016/j.bpj.2015.06.061)

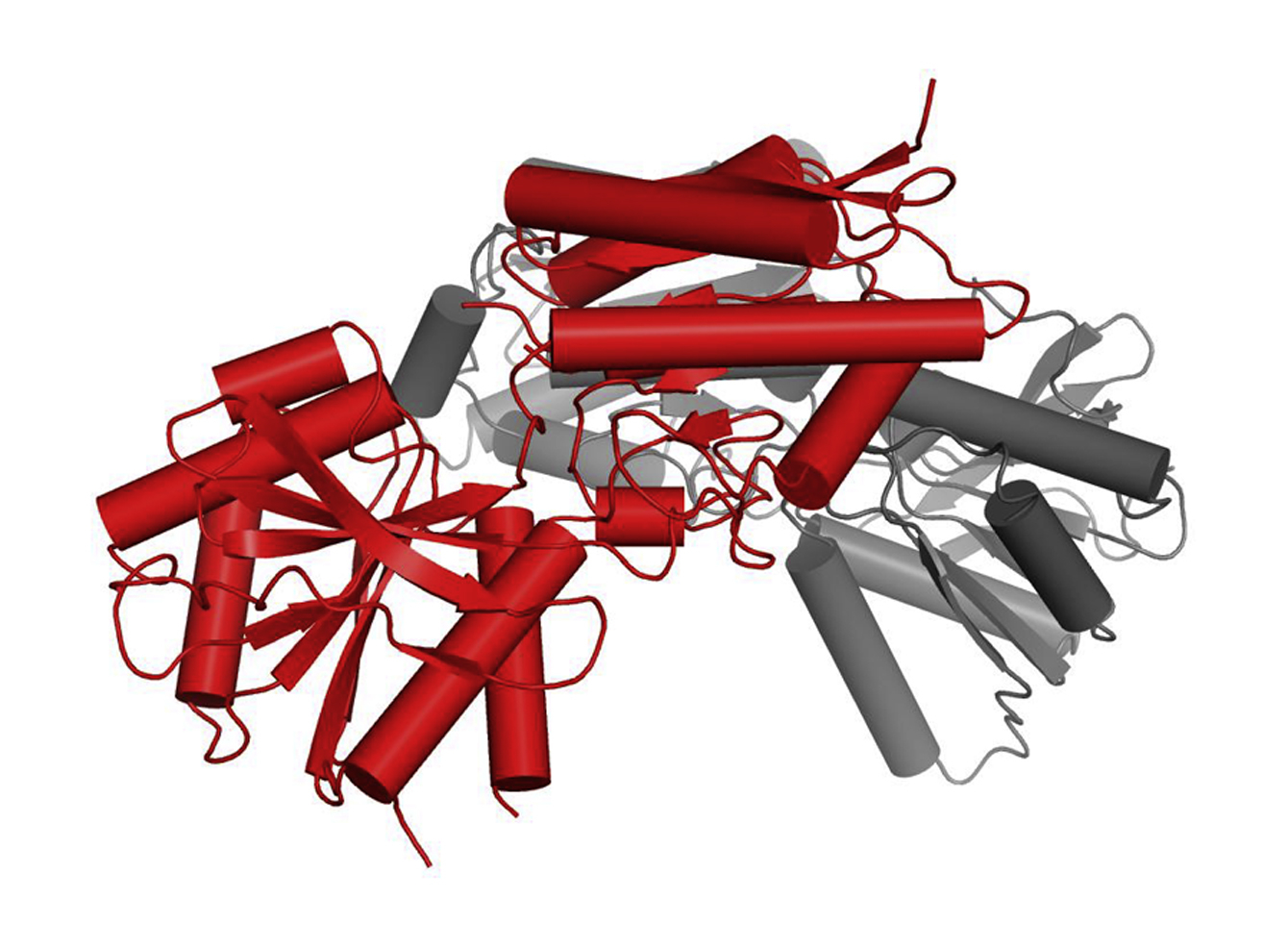

Supplement: Movie S1. GluN1/2B dimer Mode 1 showing a GluN1 cleft opening and closure coupled to dimer rearrangements — GluN1 is red and GluN2B is gray. The extent of motion was chosen to give a parallel, closed A-like state that could bind polyamines. It should be noted that GluN2B cleft motions are limited by the interaction formed between its LL and the GluN1 UL in the ifenprodil-bound starting structure. Based on functional data (31,71), we expect that it would in fact open and close in concert with GluN1. [file mmc2.jpg]

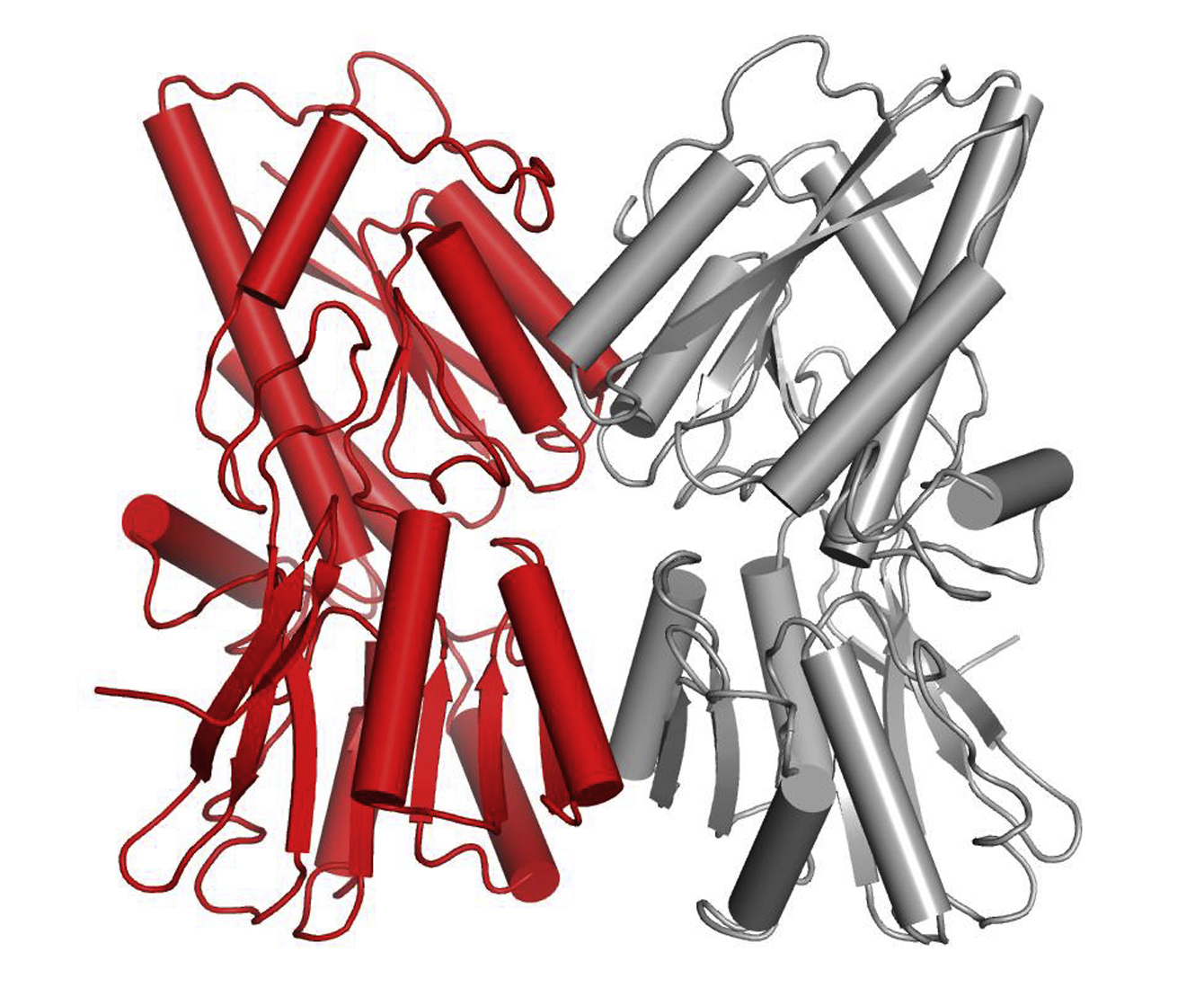

Supplement: Movie S2. GluA2 dimer Mode 1 showing a cleft opening/closure and twisting coupled to dimer rearrangements — A front view is shown, giving a view into the cleft of the gray subunit. The ANM provides information on the directionality of motions, but not on their absolute size; therefore, the extent of motion is arbitrary. [file mmc3.jpg]

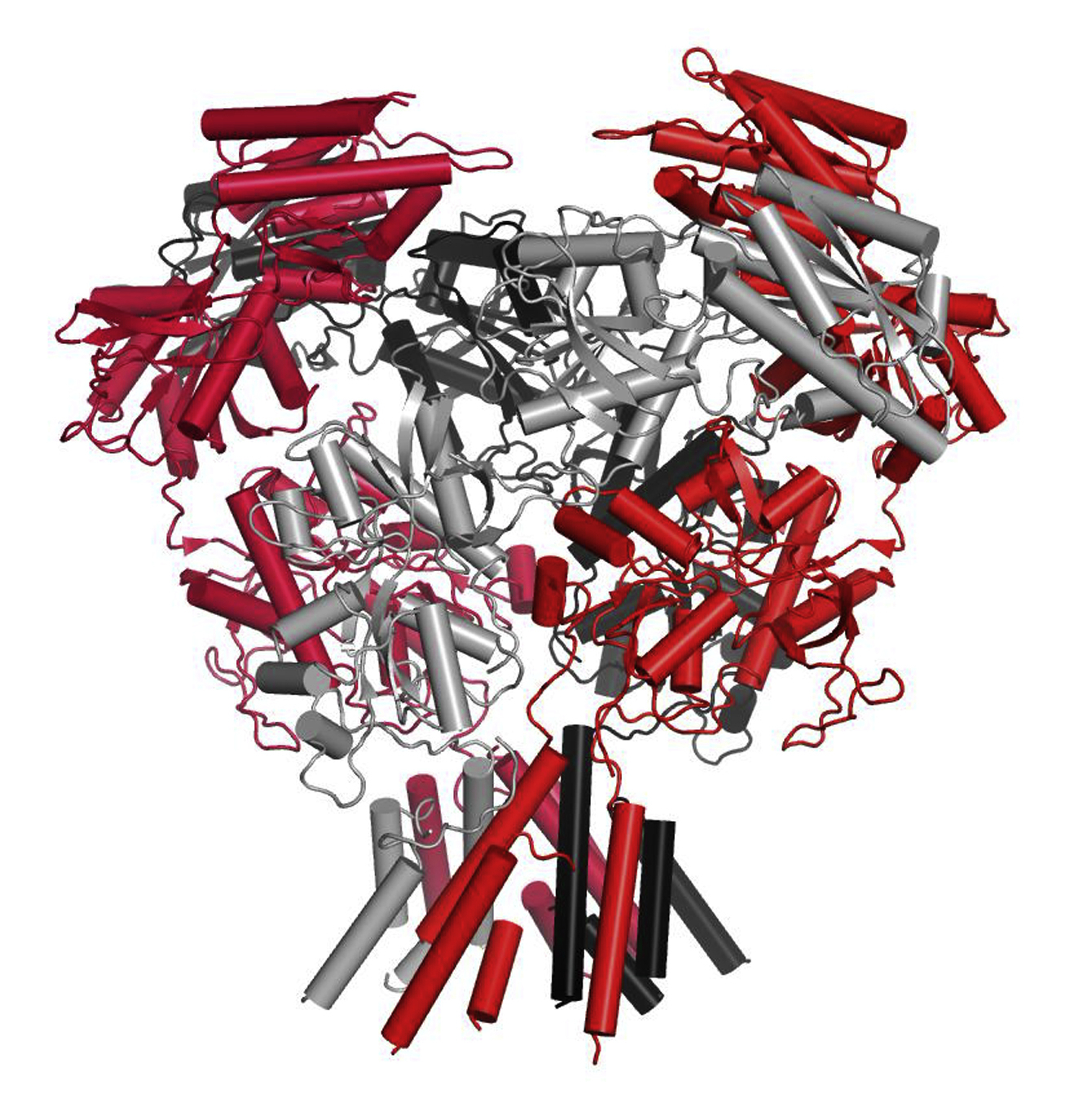

Supplement: Movie S3. Whole NMDAR Mode 6 showing GluN1 NTD cleft motions coupled to larger rearrangements — GluN1 subunits are in two reds and GluN2B subunits are in two grays. As noted in the legend for Movie S1, GluN2B cleft motions are limited in the ANM but are still expected to occur. This mode was found to be similar to AMPAR mode 7 in our most recent study (22) (see http://www.ccbb.pitt.edu/bahar/mw1.html). The ANM provides information on the directionality of motions, but not on their absolute size; therefore, the extent of motion in the movie is arbitrary. Here, the fluctuation amplitudes were selected to approximate the extent of conformational variations observed between the AMPAR and NMDAR, and to allow a clear visualization of the NTD conformational changes. [file mmc4.jpg]

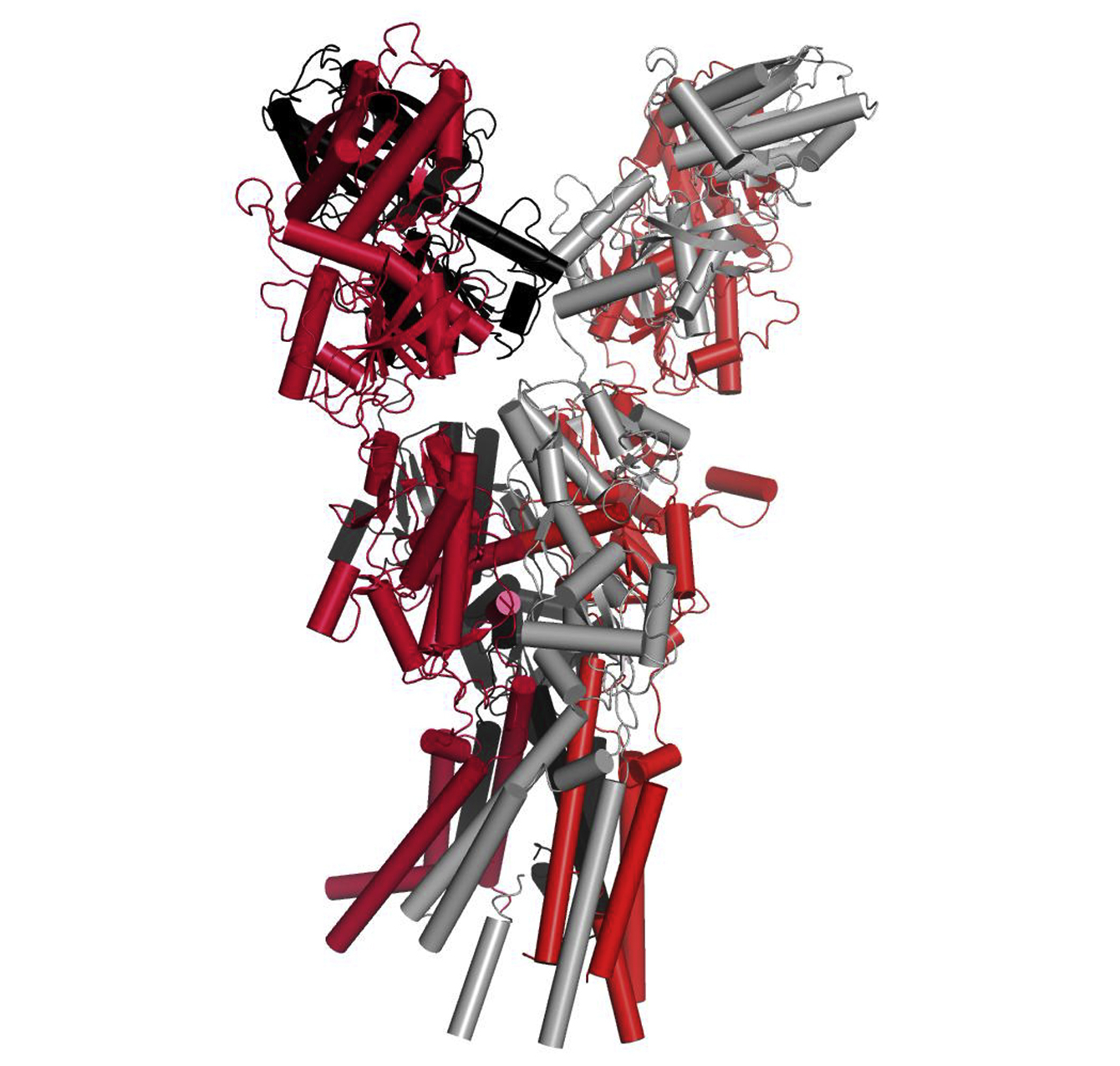

Supplement: Movie S4. Whole AMPAR Mode 11 showing NTD cleft motions coupled to larger rearrangements — Distal subunits (A/C) are in two reds and proximal subunits (B/D) are in two grays. As noted in the legend to Movie S3, the extent of motion shown is somewhat arbitrary. [file mmc5.jpg]

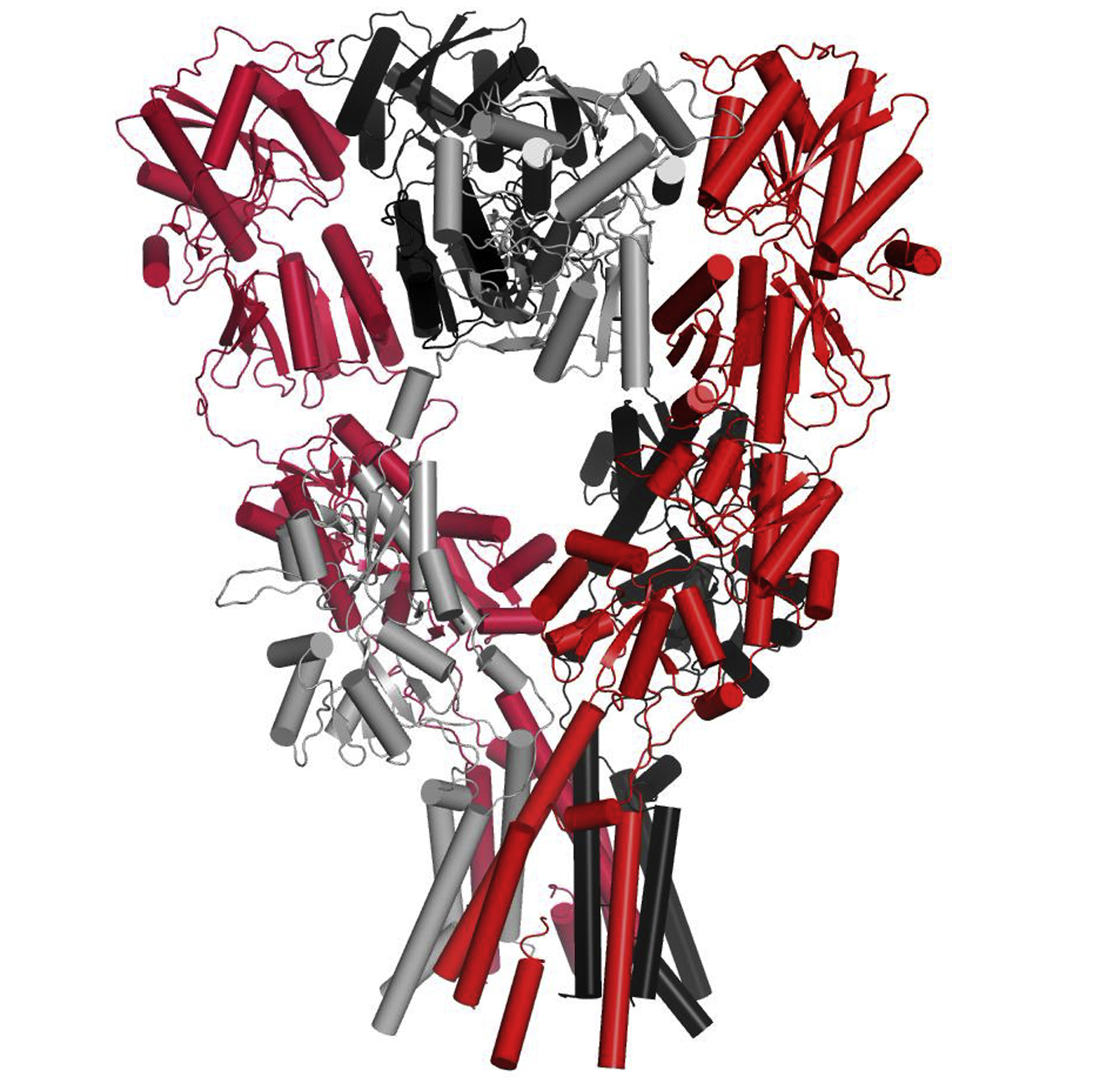

Supplement: Movie S5. Whole AMPAR Mode 12 showing NTD cleft motions coupled to larger rearrangements — Subunits are colored as in Movie S4. As noted in the legend to Movie S3, the extent of motion is somewhat arbitrary. [file mmc6.jpg]
